# Pulmonary function three to five months after hospital discharge for COVID-19: a single centre cohort study

**DOI:** 10.1038/s41598-023-27879-8

**Published:** 2023-01-13

**Authors:** Tina Krueger, Janelle van den Heuvel, Vivian van Kampen-van den Boogaart, Roel van Zeeland, D. Jannet Mehagnoul-Schipper, Dennis G. Barten, Lieve Knarren, Arno F. G. Maas, Caroline E. Wyers, Debbie Gach, Annemie M. W. J. Schols, Rosanne J. H. C. G. Beijers, Joop P. van den Bergh, Frits H. M. van Osch

**Affiliations:** 1 Department of Clinical Epidemiology, VieCuri Medical Centre, Venlo, The Netherlands; 2 Department of Pulmonology, VieCuri Medical Centre, Venlo, The Netherlands; 3 Department of Intensive Care, VieCuri Medical Centre, Venlo, The Netherlands; 4 Department of Emergency Medicine, VieCuri Medical Centre, Venlo, The Netherlands; 5 Department of Internal Medicine, VieCuri Medical Centre, Venlo, The Netherlands; 6 Department of Respiratory Medicine, NUTRIM School of Nutrition and Translational Research in Metabolism, Maastricht University Medical Centre+, Maastricht, The Netherlands; 7 Department of Internal Medicine, NUTRIM School of Nutrition and Translational Research in Metabolism, Maastricht University Medical Centre+, Maastricht, The Netherlands; 8 Department of Epidemiology, NUTRIM School of Nutrition and Translational Research in Metabolism, Maastricht University, Maastricht, The Netherlands

**Keywords:** Epidemiology, Respiratory tract diseases, Viral infection

## Abstract

Some COVID-19 survivors suffer from persistent pulmonary function impairment, but the extent and associated factors are unclear. This study aimed to characterize pulmonary function impairment three to five months after hospital discharge and the association with disease severity. Survivors of COVID-19 after hospitalization to the VieCuri Medical Centre between February and December 2020 were invited for follow-up, three to five months after discharge. Dynamic and static lung volumes, respiratory muscle strength and diffusion capacity were measured. The cohort comprised 257 patients after a moderate (n = 33), severe (n = 151) or critical (n = 73) COVID-19 infection with a median follow-up of 112 days (interquartile range 96–134 days). The main sequelae included reduced diffusion capacity (36%) and reduced maximal expiratory pressure (24%). Critically ill patients were more likely to have reduced diffusion capacity than moderate (OR 8.00, 95% CI 2.46–26.01) and severe cases (OR 3.74, 95% CI 1.88–7.44) and lower forced vital capacity (OR 3.29, 95% CI 1.20–9.06) compared to severe cases. Many COVID-19 survivors, especially after a critical disease course, showed pulmonary function sequelae, mainly DLCO impairments, three to five months after discharge. Monitoring is needed to investigate the persistence of these symptoms and the longer-term implications of the COVID-19 burden.

## Introduction

The coronavirus disease 2019 (COVID-19) has rapidly spread around the globe with more than 600 million infected people and over 6.4 million deaths so far^1^. While the majority of COVID-19 patients experience no or mild symptoms, around 20% develop serious symptoms that require hospital care^2^. Respiratory tract impairments are among the most common consequences of COVID-19^3,4^. Disease severity has been found to correlate with characteristics of the host, viral dynamics during acute infection, and the host immune response^5,6^. Especially high age, high BMI and various comorbidities including cardiovascular diseases, diabetes or cancer were associated with a severe COVID-19 course and higher mortality^7–10^.

Some of the recovered patients have reported persisting impairments—often referred to as “long-COVID”^4,11^. Daugherty et al*.* conducted a retrospective study with 193,113 participants and found increased risks for various clinical sequelae including respiratory failure after COVID-19 infection^12^. In one of the largest studies to date with 349 and 230 subjects that underwent pulmonary function testing respectively six months and 2 years after discharge, Huang et al. found pulmonary function impairments in more than 20% of COVID-19 survivors after 6 months and with no significant change after 2 years^13,14^. Impaired diffusion capacity was the most common symptom and was associated with critical courses during acute disease, which is in line with other reports^15–17^. After two years, survivors with a critical disease were at higher risk of lung diffusion impairment, residual volume and total lung capacity than their counterparts matched for age, sex and chronic pulmonary disease^14^.

The number of studies on the frequency and relevant predictors of COVID-19 related pulmonary sequelae is still limited with varying results^11,13,15–22^. Studies are restricted by small sample sizes, lack of systematic recruitment or an assessment of pulmonary function within 0–6 weeks after discharge, which impedes the distinguishability between ongoing acute inflammation and longer-term consequences^23–26^. The heterogeneity of the available data highlights the need for further studies on large cohorts to unravel the characteristics of COVID-19 sequeale. The aim of this study was to characterize the pulmonary function in a large cohort of COVID-19 survivors three to five months after hospital discharge and analyze the association with severity of the acute COVID-19 course.

## Methods

### Study design and population

This retrospective study includes a cohort of COVID-19 patients who have been admitted to the VieCuri Medical Centre in Venlo, the Netherlands, during the first (before July 1st) and second COVID wave (after July 1st) in between February and December 2020. Patients were eligible if they were older than 18 years and had a confirmed SARS-CoV-2 infection. The infection status was assessed by quantitative polymerase chain reaction using an in-house protocol from the Dutch National institute for Public Health and the Environment. All patients were invited for a standardized out-patient follow-up assessment after hospital discharge. Within this study, only data from patients that visited and completed the outpatient follow-up assessments were used.

Due to the retrospective and observational approach of the study and the acute setting in which the baseline data were gathered, a waiver of informed consent was provided by the Medical Ethical Committee of Maastricht University Medical Centre. Later, a waiver for medical ethical review was provided for the follow-up data that was gathered and patients visiting the outpatient clinic were informed about their data being used for research and had the opportunity to opt out. The study protocol and data protection impact analysis have been reviewed and approved by the medical ethics committees of the Maastricht University Medical Center (MUMC; 2020-1323).

### Measurements

For the acute phase of COVID-19 between hospital admission and discharge, both patient and hospitalization characteristics were collected. Patient characteristics including age, sex, BMI, and comorbidities were assessed by standardized questionnaires or interviews. The Charlson Comorbidity Index was calculated as described previously^27^. The variable ‘wave’ was introduced as a proxy for the different treatment procedures during the first and second wave (admission date before or after 01-07-2020). Hospitalization characteristics including duration of hospital admission, type of ward (intensive care or not) and received treatments were retrieved from medical records. Daily clinical measures were filled into the database according to the WHO—International Severe Acute Respiratory and Emerging Infections Consortium case record form. Patients were categorized into moderate, severe, and critical courses according to the WHO COVID-19 disease severity categorization^28^.

During the visit at the outpatient clinic, pulmonary function tests were performed according to the guidelines of the European Respiratory Society on a MasterScreen™ Body and MasterScreen™ PFT (PanGas, Dagmersellen) using the SentrySuite V3.0.5 software. Pre-bronchodilator spirometry was performed to assess forced expiratory volume in one second (FEV1) and forced vital capacity (FVC). Body plethysmography was used to determine total lung capacity (TLC), residual volume (RV) and maximum vital capacity (VCmax). Moreover, maximal respiratory expiratory and inspiratory pressure (PE and PI) were measured as indicator for respiratory muscle strength. Diffusion capacity of the lung for carbon monoxide (DLCO) was measured using the single-breath method. Additionally, DLCO per unit alveolar volume (KCO, sometimes referred to as DLCO/Va) was calculated. The lower limit of normal (LLN) was defined as the 5^th^ percentile according to the standardized multi-ethnic reference values for spirometry from the Global Pulmonary function initiative^29,30^. We prefer this cut-off preferred for our regression analysis over the commonly used threshold of 80% predicted due to its higher validity especially for older populations^29^. Both the % predicted values and the percentage of patients below the LLN are presented for completeness.

### Statistical analysis

Categorical variables were described by the absolute number and the corresponding percentage. Continuous variables were presented using mean and standard deviation (SD) or median and interquartile range (IQR) depending on their distribution. Normality was assessed by histograms and P-P plots. χ^2^-test or Fisher's exact test were used to compare categorical variables. For continuous variables, one-way ANOVA or Kruskal–Wallis were used as appropriate followed by Tukey or Wilcoxon test with Holm correction for post-hoc pairwise comparisons, respectively.

Multivariable linear and logistic regression models were built for the association of severity as independent variable and DLCO % predicted and the percentage of the parameter of interest < LLN as respective dependent variables. We considered the variables age, sex, BMI, pulmonary comorbidities and days between discharge and follow-up as potential confounders. The models were build in a forward model building approach. Variables remained in the respective final model when they introduced a 10% change-in-estimate or a significant increase in model fit as expressed by log likelihood or R^2^.

The study was sufficiently powered for showing an association between disease severity and the pulmonary outcomes of interest with a minimal effect size 0.07 considering an alpha of 0.05 and power of 0.80. All statistical tests were performed in R (V4.0.2) with a two-sided significance threshold of 5% and GPower (V3.1) was used for the power calculation^31^.

### Ethics approval

This study was approved by the institutional review board of VieCuri Medical Centre. Due to the retrospective and observational approach of the study, a waiver of informed consent was provided.

### Consent to participate

Participation was voluntary. Data were collected retrospectively. Patients had the opportunity to opt out and if they did, data were not used.

## Results

In total, 545 PCR confirmed COVID-19 patients were hospitalized between February and December 2020 in the VieCuri Medical Centre. Of these patients, 171 died during hospital admission or before the scheduled follow-up visit (Fig. S1). Of the 374 survivors, 278 (75%) attended the outpatient follow-up visit after hospital discharge with a median interval of 112 days (IQR 96–134 days). Most of the patients that did not attend the follow-up visit were living outside the region of the VieCuri hospital (16%). These patients were transferred to VieCuri Medical Centre for admission because of limited capacity in their regional hospital (mostly during the start of the second wave). Therefore, these patients were followed up by their regional hospital. Of the 278 patients with follow-up assessment, complete pulmonary function, demographic and treatment data were available from 257 patients that were included in the current analysis (Fig. S1).

Baseline characteristics are summarized for the total group and according to disease severity (Table 1). The median age of the cohort was 67 years (IQR 59–75 years) and 102 patients (41%) were female. The disease course was severe in 151 (59%) and critical in 73 patients (28%). At least one comorbidity was present in 83% patients, with hypertension as the most common one (47%). In total, 22% of the patients had a history of pulmonary diseases including chronic obstructive pulmonary disease (COPD) or asthma. The median length of the hospital stay was 6 days (IQR 3–11 days) and 18% of the patients were admitted to intensive care unit (ICU). Most patients received supplemental oxygen (84%) and 27% of the patients were mechanically ventilated including 12% with non-invasive and 15% with invasive ventilation. Information on the pressure of arterial oxygen (PaO2) and the fractional inspired oxygen (FiO2) were only available for a limited number of patients (66 and 68 of 257, respectively) None of the patients received extracorporeal life support.

**Table 1:** Characteristics of enrolled patients at baseline and during the hospital stay according to disease severity.

Patient characteristics	Total	Moderate	Severe	Critical	p-value
Total	N = 257	N = 33	N = 151	N = 73	
Age in years, mean ± SD	66 ± 12	67 ± 14	67 ± 12	64 ± 10	0.222
Male	152 (59%)	18 (55%)	90 (60%)	44 (60%)	0.843
BMI, mean ± SD	28 ± 5	27 ± 4	28 ± 5	29 ± 5	0.215
Comorbidities					
Hypertension	121 (47%)	16 (48%)	69 (46%)	36 (49%)	0.866
Chronic pulmonary diseases	57 (22%)	8 (24%)	36 (24%)	13 (18%)	0.568
COPD only	36 (14%)	4 (12%)	24 (16%)	8 (11%)	0.575
Asthma only	9 (4%)	2 (6%)	5 (3%)	2 (3%)	0.677
COPD and Asthma	12 (5%)	2 (6%)	7 (5%)	3 (4%)	0.907
Chronic cardiac diseases	61 (24%)	12 (36%)	34 (23%)	15 (21%)	0.179
Rheumatologic disorder	36 (14%)	5 (15%)	22 (15%)	9 (12%)	0.884
Auto-immune disorder	30 (12%)	3 (9%)	18 (12%)	9 (12%)	0.881
Diabetes	63 (25%)	9 (27%)	37 (25%)	17 (23%)	0.907
Malignant neoplasms	22 (9%)	6 (18%)	10 (7%)	6 (8%)	0.098
CCI, Median (IQR)	3 (2–4)	3 (2–7)	3 (2–5)	3 (1–4)	0.418
Hospital stay					
Days from symptom onset to admission, Median (IQR)	8 (6–11)	8 (6–11)	9 (7–12)	7 (5–10)	0.095
Days from admission to discharge, Median (IQR)	6 (3.25–11)	2 (1–4)	6 (4–9)***	10 (5–30.5)***^###^	< 0.005
ICU admission	45 (18%)	0 (0%)	5 (3%)	40 (55%)***^###^	< 0.005
Days from discharge to follow-up assessment, median (IQR)	112 (96–134)	126 (104–142)	112 (97–132)	104 (90–135)	0.107
Pulmonary embolisms	17 (7%)	1 (3%)	8 (5%)	8 (11%)	0.224
FiO2, median (IQR)	0.36 (0.28–0.4)	0.28 (0.28–0.28)	0.32 (0.28–0.39)	0.44^###^ (0.36–0.8)	< 0.005
PaO2/FiO2, median (IQR)	26 (20–30)	29 (29–29)	27 (22–31)	19 (16–29)	0.046
ARDS	4 (2%)	0 (0%)	0 (0%)	4 (5%)^#^	n.a
Nasal high flow	10 (4%)	0 (0%)	0 (0%)	10 (14%)	n.a
Non-invasive ventilation	30 (12%)	0 (0%)	0 (0%)	30 (41%)	n.a
Invasively ventilated	39 (15%)	0 (0%)	0 (0%)	39 (53%)	n.a
Proning	20 (8%)	0 (0%)	0 (0%)	20 (27%)***^###^	n.a
Vasopressors/inotropes	39 (15%)	0 (0%)	0 (0%)	39 (53%)	n.a
Treatment in hospital					
Chloroquine	103 (40%)	2 (6%)	53 (35%)***	48 (66%)***^###^	< 0.005
Dexamethasone	51 (20%)	5 (15%)	37 (25%)	9 (12%)	0.078
Anticoagulants	30 (12%)	7 (21%)	15 (10%)	8 (11%)	0.173
Antibiotics	177 (69%)	16 (48%)	98 (65%)	63 (86%)***^###^	< 0.005
Follow-up treatment					
Revalidation therapy	48 (19%)	2 (6%)	13 (9%)	33 (45%)***^###^	< 0.005

Continuous parameters are presented as mean ± SD and analyzed with one-Way Anova with Tukey post-hoc test or presented as median (IQR) and analyzed with Kruskal–Wallis test with Wilcoxon post hoc test. Chi-squared test and Fisher’s exact were applied to categorical variables as appropriate. p-values: *< 0.05, **< 0.01, ***< 0.005 compared to the moderate reference group, ^#^< 0.05, ^##^< 0.01, ^###^< 0.005 compared to the severe reference group. *FiO2* fractional inspired oxygen, *PaO2* oxygen partial pressure, *ARDS* Acute Respiratory Distress Symptom, *BMI* Body mass index, *CCI* Charlson Comorbidity Index, *ICU* Intensive care unit.

Baseline characteristics and comorbidities were not significantly different between the severity groups (Table 1). Patients with a critical disease course were more often treated at the ICU, had a longer hospital stay and received antiviral or antibiotic treatment more frequently. The time between discharge and follow-up assessment was similar for all severity groups. Chloroquine, which was used only during the first wave, was more frequently administered to patients with severe and critical courses. Dexamethasone was used during the second wave only, but administration was not different between the disease severity stages. Patients infected during the first and second wave differed in received treatments as a result of changes in medical guidelines. Additionally, fewer patients had a critical disease course during the second wave (Appendix, Supplementary Table 1).

Pulmonary function assessment was performed at a median of 112 (96–134) days after hospital discharge (Table 2). Patients with critical COVID-19 showed significantly lower DLCO and TLC % predicted values compared to severely and moderately diseased patients as well as lower RV % predicted values compared to the severe group. We did not observe any significant differences in other pulmonary outcomes between the different severity groups. We found the highest number of abnormalities for DLCO (36%), PE (24%) and RV (16%). One out of five patients with moderate illness and half of the patients with critically illness had DLCO impairment.

**Table 2:** Mean % predicted values and proportion of patients with abnormal pulmonary function (< LLN) at follow-up.

		Total	Moderate	Severe	Critical	p-value
Total		N = 257	N = 33	N = 151	N = 73	
FEV1	% pred	94 ± 22	93 ± 19	95 ± 22	94 ± 23	0.899
	< LLN	34/243 (14%)	5/30 (17%)	17/144 (12%)	12/69 (17%)	0.493
FVC	% pred	96 ± 18	97 ± 14	97 ± 18	93 ± 19	0.231
	< LLN	24/242 (10%)	2/30 (7%)	9/143 (6%)	13/69 (19%)^##^	0.014
FEV/FVC	% pred	95 ± 16	93 ± 13	94 ± 17	99 ± 16	0.109
	< LLN	28/242 (12%)	3/30 (10%)	20/143 (14%)	5/69 (7%)	0.342
VCmax	% pred	104 ± 18	105 ± 16	106 ± 18	100 ± 20	0.106
	< LLN	17/236 (7%)	2/28 (7%)	6/140 (4%)	9/68 (13%)	0.064
RV	% pred	95 ± 27	99 ± 24	98 ± 28	87 ± 23^#^	0.015
	< LLN	37/232 (16%)	3/27 (11%)	18/138 (13%)	16/67 (24%)	0.106
TLC	% pred	97 ± 15	100 ± 12	98 ± 15	91 ± 16*^###^	< 0.005
	< LLN	40/231 (17%)	4/27 (15%)	17/137 (12%)	19/67 (28%)^##^	0.017
PE	% pred	93 ± 39	97 ± 39	91 ± 41	94 ± 35	0.700
	< LLN	56/232 (24%)	4/28 (14%)	40/135 (30%)	12/69 (17%)	0.066
PI	% pred	100 ± 39	100 ± 31	97 ± 40	107 ± 41	0.198
	< LLN	21/230 (9%)	1/28 (4%)	16/133 (12%)	4/69 (6%)	0.191
DLCO SB	% pred	78 ± 20	85 ± 14	80 ± 18	71 ± 22***^###^	< 0.005
	< LLN	84/233 (36%)	6/28 (21%)	41/137 (30%)	37/68 (54%)***^###^	< 0.005
kCO	% pred	88 ± 19	92 ± 15	89 ± 18	84 ± 22	0.107
	< LLN	50/233 (21%)	3/28 (11%)	27/137 (20%)	20/68 (29%)	0.094

Continuous parameters are presented as mean ± SD and analyzed using one-way ANOVA with Tukey post-hoc test or presented as median (IQR) and analyzed using Kruskal–Wallis test with Wilcoxon post hoc test. Chi-squared test and Fisher’s exact were applied to categorical variables as appropriate. *FUP* follow-up. p-values: *< 0.05, **< 0.01, ***< 0.005 compared to the moderate reference group, ^#^< 0.05, ^##^< 0.01, ^###^< 0.005 compared to the severe reference group.

The odds of having a pulmonary function < LLN was significantly increased in patients that had critical COVID-19 compared to those with severe COVID-19 for FVC and TLC and to patients with a moderate and severe COVID-19 for DLCO. There were no differences in any pulmonary function parameter between first and second wave patients, although the latter had a longer time between hospital discharge and follow up assessment (Appendix, Supplementary Table 2).

To account for potential confounding, we analyzed the association of COVID-19 severity and pulmonary function impairment in a multivariable logistic regression model adjusting for age, sex, BMI, pulmonary comorbidities and time between discharge and follow-up (Table 3). After adjustment, patients with critical COVID-19 showed significantly higher odds of DLCO impairment (OR 8.00, 95% CI 2.46–26.01 compared to moderate; 3.74, 1.88–7.44 compared to severe) and FVC (OR 3.29, 95% CI 1.2–12.73 compared to severe), while TLC impairment was not significantly different between severity groups after multivariable adjustment. ‘Wave’, as a proxy for different patient handling and treatment was not associated with any pulmonary function parameter.

**Table 3:** Unadjusted and multivariable-adjusted logistic regression.

		Unadjusted		Fully adjusted	
		OR (95% CI)	p	OR	p
FEV1	Severe vs moderate	0.67 (0.23–1.98)	0.468	0.65 (0.19–2.29)	0.506
	Critical vs moderate	1.05 (0.34–3.31)	0.930	1.49 (0.39–5.71)	0.563
	Critical vs severe	1.57 (0.71–3.51)	0.269	2.27 (0.87–5.98)	0.095
FVC	Critical vs severe	3.46** (1.4–8.55)	0.007	3.29* (1.2–9.06)	0.021
TLC	Critical vs severe	2.79** (1.34–5.83)	0.006	2.25 (1–5.07)	0.050
DLCO	Severe vs moderate	1.57 (0.59–4.15)	0.367	2.14 (0.71–6.42)	0.174
	Critical vs moderate	4.38** (1.58–12.15)	0.005	8.00*** (2.46–26.01)	< 0.005
	Critical vs severe	2.79*** (1.53–5.1)	< 0.005	3.74*** (1.88–7.44)	< 0.005

The adjusted logistic regression model included age, sex, BMI, pulmonary comorbidities, and time between discharge and follow-up. Regression coefficients were only presented if number of events per stratum was higher than five.

p-values: *< 0.05, **< 0.01, ***< 0.005.

**Figure 1:**
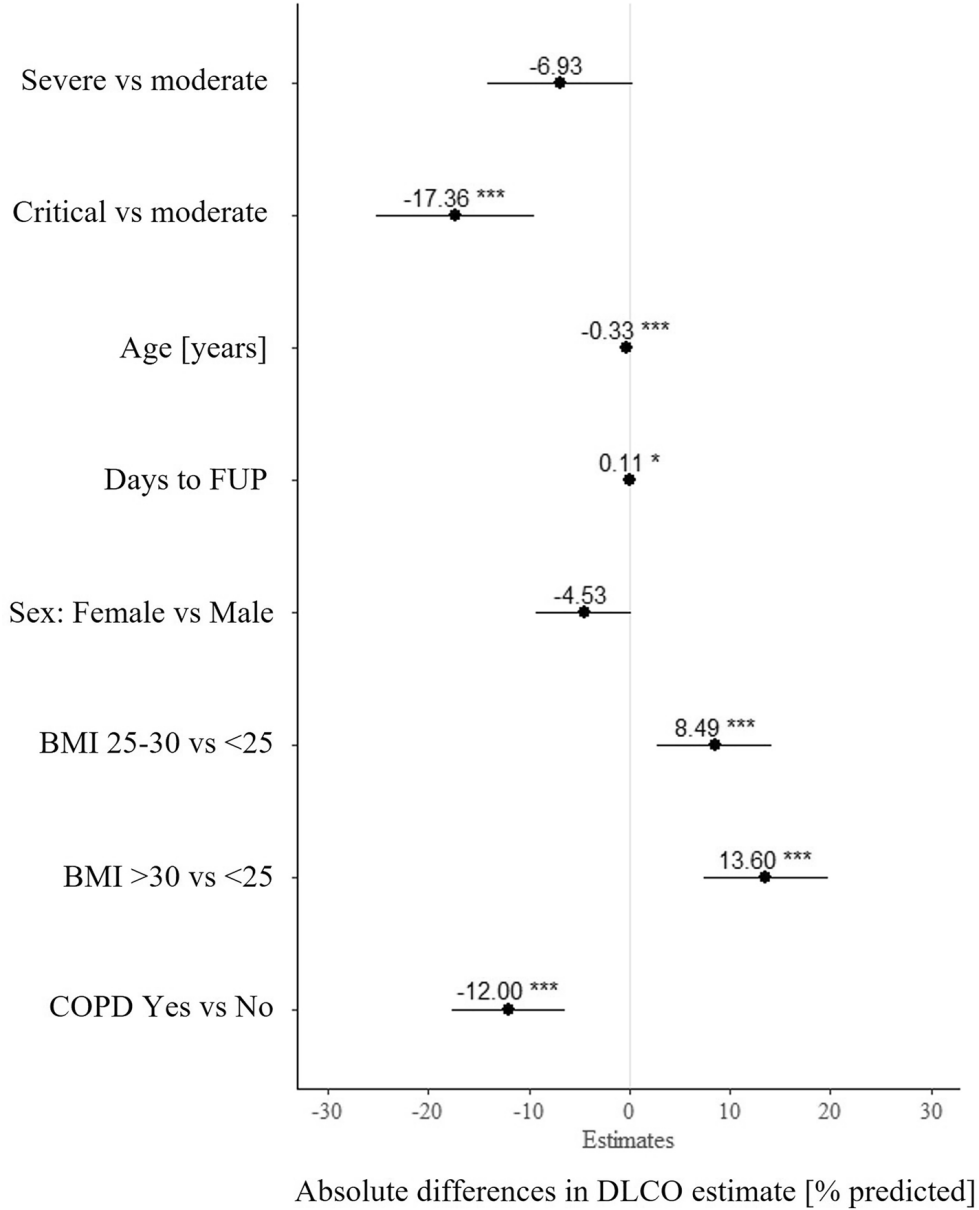
Multivariable adjusted linear regression model for DLCO % predicted values.

Figure 1 shows the estimated mean differences in DLCO% predicted in a multivariable adjusted linear regression analysis including disease severity, age, gender, BMI, COPD, and time span between hospital discharge and follow-up assessment. Patients with a critical acute disease had on average 17% lower DLCO% predicted values compared to a moderate disease after adjustment for age, sex, BMI, pre-existing COPD and the time between discharge and follow-up. Besides a critical disease course, high age, being female and pre-existing COPD were associated with low DLCO values. A higher BMI was correlated with higher DLCO% predicted values. Patients with a longer time interval between hospital discharge and follow-up assessment had a significantly higher diffusion capacity.

We performed a subgroup analysis for DLCO impairment between invasively ventilated and non-invasively ventilated patients within the critical group to gain insights about potential post-ventilation effects. Critically ill patients had higher odds for impaired DLCO but not for impaired lung volumes or PE if they had been invasively ventilated during their hospital stay. Respectively, the mean predicted values were lower for DLCO and TLC in invasively ventilated patients (Table 4).

**Table 4:** Association of invasive ventilation (n = 39) versus non-invasive ventilation (n = 30) with the proportion of patients with anomalies in pulmonary function (< LLN) and % predicted values within patients after critical disease.

	OR (95% CI) of anomalies	p-value	Mean difference [% pred]	p-value
FEV1	1.21 (0.34–4.29)	1	− 5.28 (− 16.66 to 6.1)	0.357
FVC	0.97 (0.29–3.27)	1	− 7.02 (− 16.32 to 2.28)	0.136
TLC	1.51 (0.51–4.51)	0.586	− 7.95* (− 15.55 to – 0.34)	0.041
PE	0.81 (0.23–2.81)	0.760	− 0.05 (− 17.12 to 17.02)	0.995
DLCO	3.6* (1.31–9.9)	0.015	− 13.7* (− 24.03 to – 3.36)	0.010

OR with their corresponding p-values were derived by Wald-test and mean differences by two-sample Student’s t-test.

p-values: *< 0.05.

## Discussion

This study aimed to characterize the pulmonary function of patients who had been admitted to the hospital due to COVID-19 three to five months after hospital discharge and test the association with disease severity. We found diffusion capacity and respiratory muscle strength impairment among the most common pulmonary anomalies after hospital discharge with a median follow-up interval of 112 days. Our results for the prevalence of pulmonary function impairments are in line with those from Huang et al. who conducted one of the largest studies so far with pulmonary function tests in 349 patients 6 months after symptom onset. Other studies found similar results after 3 to 4 months after hospital discharge with abnormalities in DLCO for 16–88%, in FVC for 11–37% and in TLC for 7–53% of the included patients^16,32–34^. The variation in prevalence could be explained by differences in the study population and the inconsistent definition of normality for pulmonary function parameters based on the LLN or the 80% predicted.

We found an association between acute disease severity and lower DLCO, FVC and TLC, which is in line with other studies^17,35^. These parameters were also found to be impaired in survivors two years after hospital discharge compared to matched controls^14^. Contrary to Chun et al., we did not find an association between disease severity and FEV1 which might be explained by the different study population that also included non-hospitalized patients^36^. However, despite our relatively large sample size, the power of our study might not suffice to find such associations of smaller magnitude considering the limited number of subjects in the moderate subgroup.

The association between disease severity and DLCO remained after correcting for age, sex, BMI, pulmonary comorbidities, and the variation in time between discharge and follow-up assessment. We found a significant association between the time covariate and DLCO which might hint towards ongoing pulmonary recovery. In a longitudinal study by Wu et al*.* with similar results after 3 months, DLCO values had significantly improved 12 months after discharge whereas Huang et al*.* could not observe pulmonary function improvement in a 2 year follow-up^14,33^. A small number of other studies showed an association between female sex and DLCO or other pulmonary abnormalities, which is in line with our observation^14,33,37,38^. However, our study and the regression model are not tailored to reveal the effect of sex on pulmonary function at follow-up and future studies are needed to primarily address this question.

A subgroup analysis within the critical group revealed that mechanical ventilation is highly correlated with impaired DLCO indicating that our results could at least partly be explained by post ventilation effects on the lung as previously reported in patients without pulmonary disease^39^. However, similar results have been found by Mo et al. in a cohort excluding mechanically ventilated patients suggesting an independent association between acute COVID-19 severity and DLCO impairment that is not mediated by post ventilation effects^25^.

Further research is needed that focusses on the temporal development of long-term pulmonary symptoms in patients after hospitalization for COVID-19. In a comprehensive health assessment of 124 patients with a very similar pattern of DLCO impairment, the majority had functional impairment, fatigue and poor qualtiy of life, independent of disease severity^14^. Similar tests and CT imaging should be included in future studies to better understand structural changes and functional impact.

Our study has several strengths and limitations. To our knowledge, there are only a few studies of this sample size that invited all patients after hospitalization due to COVID-19 for systematic pulmonary function tests. Moreover, we defined abnormal pulmonary function based on the lower limit of normal, which has been shown to be less prone to misclassifications, especially in older populations^29^. Our data will add power to future meta-analyses reviewing the respiratory function after COVID-19 infection as the currently available reviews included a limited number of studies with high heterogeneity and low or medium quality^24,40^. The main limitation of our study is the lack of baseline values for pulmonary function prior to COVID-19 hospitalization, which might introduce a positive bias if patients, e.g. with COPD or asthma-COPD overlap syndrome, already had poor pulmonary function at baseline^41^. We accounted for this by adjusting for chronic pulmonary diseases in the regression model and still found substantial associations. Furthermore, not considering patients admitted to our hospital from another region that were missing at random, less than 5% of patients did not show up at follow-up measurements for various reasons, of which some could have had significantly impaired lung function. Nevertheless, the numbers were so small that this will not have impacted the association measures in a relevant way.

In conclusion, our study showed that pulmonary abnormalities, especially reduced diffusion capacity, are common in discharged COVID-19 patients at three to five months after hospital discharge. More research is needed to better understand the characteristics, time course and risk factors of long-term pulmonary COVID-19 sequelae. Especially patients with critical cases should be closely monitored and considered for routine functional and structural pulmonary follow-up as well as rehabilitation programs.

## Supplementary Information

Supplementary Information.

## Data Availability

Data are available upon request to the corresponding author.

## Code availability

Scripts are available upon request to the corresponding author.
