# Supplementary material for: Pulmonary function three to five months after hospital discharge for COVID-19: a single centre cohort study
Source: Sci Rep. 2023 Jan 13;13:681. doi: 10.1038/s41598-023-27879-8 (PMC9839688; doi:10.1038/s41598-023-27879-8)
Supplement: Supplementary file 1 — Supplementary Information. [file 41598_2023_27879_MOESM1_ESM.docx]

# Appendix


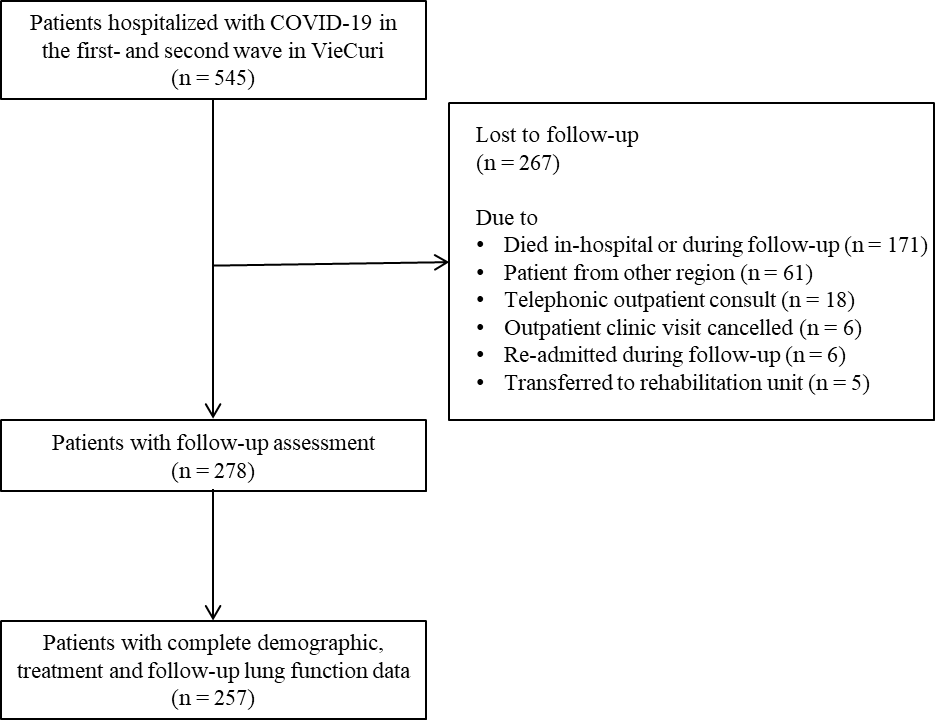


**Fig. S1** Flowchart outlining the number of hospitalized patients and proportion of patients for whom pulmonary function was measured after hospital discharge with a median follow-up of 112 days (IQR 96-134 days)

**Supplementary Table 1** Comparison of baseline and hospital stay characteristics of 1^st^ and 2^nd^ wave patients

| **Patient characteristics** | **1^st^ Wave** | **2^nd^ Wave** | **p-value** |
| --- | --- | --- | --- |
| Total | N = 188 | N = 69 |  |
| Age in years, Mean ±SD | 66±11 | 66±13 | 0.797 |
| Male | 114 (61%) | 38 (55%) | 0.421 |
| BMI, Mean ±SD | 28±5 | 28±4 | 0.999 |
| **Comorbidities** |  |  |  |
| Hypertension | 93 (49%) | 28 (41%) | 0.206 |
| Chronic pulmonary diseases | 42 (22%) | 15 (22%) | 0.918 |
| Chronic cardiac diseases | 49 (26%) | 12 (17%) | 0.148 |
| Rheumatologic disorder | 27 (14%) | 9 (13%) | 0.787 |
| Auto-immune disorder | 22 (12%) | 8 (12%) | 0.981 |
| Diabetes | 43 (23%) | 20 (29%) | 0.313 |
| Malignant neoplasms | 19 (10%) | 3 (4%) | 0.144 |
| CCI, Median (IQR) | 3 (1.75-4) | 3 (2-7) | 0.352 |
| **Hospital stay** |  |  |  |
| Days from symptom onset to admission, Median (IQR) | 8 (7-12) | 9 (5-10) | 0.449 |
| Days from admission to discharge,  Median (IQR) | 6 (4-13) | 6 (3-8.25) | 0.264 |
| Severity: Moderate | 22 (12%) | 11 (16%) | 0.011 |
| Severe | 103 (55%) | 48 (70%) |  |
| Critical | 63 (34%) | 10 (14%) |  |
| ICU admission | 33 (18%) | 12 (17%) | 0.976 |
| **Treatment** |  |  |  |
| Chloroquine | 103 (55%) | 0 (0%) | <0.005 |
| Dexamethasone | 0 (0%) | 51 (74%) | <0.005 |
| Anticoagulants | 24 (13%) | 6 (9%) | 0.448 |
| Antibiotics | 135 (72%) | 42 (61%) | 0.164 |

Continuous parameters are presented as mean±SD and analyzed with Student’s t-test or presented as median (IQR) and analyzed with Wilcoxon test. Chi-squared test and Fisher’s exact were applied to categorical variables as appropriate.

**Supplementary Table 2** Results of follow-up pulmonary function assessment for 1^st^ and 2^nd^ wave patients

|  |  | **1^st^ Wave** | **2^nd^ Wave** | **p-value** |  |
| --- | --- | --- | --- | --- | --- |
| Total |  | N = 188 | N = 69 |  |  |
| Days to FUP |  | 120 (103-139) | 94 (90-100) | <0.005 |  |
| FEV1 | % | 94±22 | 94±21 | 0.992 |  |
|  | < LLN | 26/182 (14%) | 8/61 (13%) | 0.988 |  |
| FVC | % | 96±19 | 95±16 | 0.496 |  |
|  | < LLN | 18/182 (10%) | 6/60 (10%) | 1 |  |
| FEV/FVC | % | 95±17 | 97±13 | 0.476 |  |
|  | < LLN | 21/181 (12%) | 7/61 (11%) | 1 |  |
| VC MAX | % | 105±18 | 102±18 | 0.266 |  |
|  | < LLN | 13/176 (7%) | 4/60 (7%) | 1 |  |
| RV | % | 95±26 | 94±30 | 0.928 |  |
|  | < LLN | 26/173 (15%) | 11/59 (19%) | 0.653 |  |
| TLC | % | 97±15 | 95±16 | 0.406 |  |
|  | < LLN | 25/172 (15%) | 15/59 (25%) | 0.088 |  |
| PE | % | 93±38 | 93±42 | 0.900 |  |
|  | < LLN | 41/175 (23%) | 15/57 (26%) | 0.792 |  |
| PI | % | 101±39 | 98±40 | 0.565 |  |
|  | < LLN | 14/174 (8%) | 7/56 (12%) | 0.459 |  |
| DLCO SB | % | 78±21 | 78±15 | 0.929 |  |
|  | < LLN | 66/176 (38%) | 18/57 (32%) | 0.515 |  |
| kCO | % | 87±21 | 90±14 | 0.396 |  |
|  | < LLN | 43/176 (24%) | 7/57 (12%) | 0.079 |  |
| Continuous parameters are presented as mean±SD and analyzed using one-Way ANOVA with Tukey post-hoc test or presented as median (IQR) and analyzed using Kruskal-Wallis test with Wilcoxon post hoc test. Chi-squared test and Fisher’s exact were applied to categorical variables as appropriate. | | | | | |
